# The Effectiveness of Osteoporosis Screening and Treatment in the Midwest

**DOI:** 10.1177/2151459318765844

**Published:** 2018-03-29

**Authors:** Dharmik Patel, John R. Worley, David A. Volgas, Brett D. Crist

**Affiliations:** 1 School of Medicine, University of Missouri, Columbia, MO, USA; 2 Department of Orthopaedic Surgery, University of Missouri, Columbia, MO, USA

**Keywords:** osteoporosis, bone mineral density, dual-energy X-ray absorptiometry, questionnaires, patient education

## Abstract

**Introduction:**

With osteoporosis on the rise across the United States, the goal of this prospective study is to determine the effectiveness of our Midwest level-1 trauma center in diagnosing, treating, and educating osteoporosis patients after fracture with the use of questionnaires. Secondarily, we aimed to identify barriers that prevent our patients from complying with bone health recommendations.

**Methods:**

One hundred participants (≥55 years) were given 2 questionnaires (Fracture Risk Assessment Tool and a study-specific questionnaire) that were administered during the patient’s visit to the orthopedic trauma clinic. A group of patients diagnosed with osteoporosis was compared to a group of patients not diagnosed with osteoporosis. Statistical analyses were performed using SPSS 24 (IBM Corp, Chicago, Illinois).

**Results:**

Patients who had been diagnosed with osteoporosis were significantly older (72.7 vs 66.5, *P* = .009) and more were women (86.2% vs 66.2%, *P* = .043). Significantly, fewer patients without the diagnosis of osteoporosis had a history of fragility fracture (56.3%) compared to 92.9% of those diagnosed with osteoporosis (*P* < .001). Of those with dual-energy X-ray absorptiometry (DXA) recommended by a healthcare provider, 20 (55.6%) of those without the diagnosis of osteoporosis and 13 (52%) of those with the diagnosis of osteoporosis had DXA screening before their fragility fracture (*P* = .499). More patients diagnosed with osteoporosis (93.1%) were taking calcium and vitamin D supplementation compared to 66.2% of those without the diagnosis of osteoporosis (*P* = .005). Only 37.9% of patients with the diagnosis of osteoporosis were receiving US Food and Drug Administration–approved medications for the management of their disease.

**Discussion:**

In patients without previous osteoporosis diagnosis, 59 (83.1%) of the 71 claimed that they did not receive any preventative education about osteoporosis, while 21 (72.4%) of the 29 patients with the diagnosis of osteoporosis claimed that they did not receive a preventative education (*P* = .165). Both groups lacked optimum diagnosis, treatment, and education of osteoporosis.

**Conclusion:**

Our study highlights the need for a deliberate effort of a multidisciplinary team to focus efforts in all stages of osteoporosis management.

## Introduction

Osteoporosis will be one of the leading causes of morbidity by the year 2020.^1^ There were 10.2 million older adults with osteoporosis and 43.4 million with low bone mass in the United States in 2010.^2^ These numbers are expected to grow to 13.6 million people with osteoporosis and 57.8 million people with low bone mass by 2020.^2^ Although it is one of the leading causes of significant morbidity and increased mortality, osteoporosis-related fractures can be preventable if osteoporosis is diagnosed and treated early.

A fracture is the major clinically relevant consequence of having a low bone mineral density (BMD). A hip fracture is the most debilitating of the fragility fractures and has 12-month mortality of up to 36% on average but even higher (up to 58%) in nursing home residents.^3–8^ These patients are also more likely to have a second hip fracture within the next 5 years.^9^ Additionally, fragility fractures are an enormous economic burden with an estimated cost of $17 billion for more than 2 million incident fractures in the United States in 2005, and the cost is projected to rise by almost 50% by 2025.^1,10^ After a fragility fracture, the goal is to minimize the risk for secondary fracture by evaluating patients for osteoporosis and by providing them appropriate treatment and education. Unfortunately, the medical community has failed to provide appropriate screening, education, or treatment for osteoporosis leading to worse quality of life after fragility fracture and increased financial burden.^6,11^ Therefore, it is essential to investigate the effectiveness of patient education, prevention, and treatment of these high-risk fracture patients.

The barriers to successful identification and management of osteoporosis include lack of appropriate screening and diagnosis, evaluating medication side effects and cost, and patient education. Poor patient education affects treatment and lifestyle modification compliance. The majority of primary care physicians perceive cost and adverse effects of the medication as the 2 major barriers to osteoporosis treatment.^12^ Patient education is essential in chronic and clinically silent diseases like osteoporosis. Treatment attempts to prevent further resorption of the bone and slow the progression of eventual loss of microarchitectural bone structure. Hence, having patients who are compliant with the treatment recommendation—as well as lifestyle change recommendations—is essential.

Orthopedic surgeons can be the most effective care providers to at least start the conversation about osteoporosis. They treat patients with fragility fractures and have the opportunity to identify patients with clinically significant osteoporosis.^13,14^ Our academic Midwest American College of Surgeons level-1 orthopedic trauma service has been proactive in recommending dual-energy X-ray absorptiometry (DXA) screening and follow-up for the management of patients who sustain a fragility fracture. However, there is a shortage of data about how active our geographic region has been with the management of osteoporosis in at-risk patients before the event of a fragility fracture. The purpose of this cross-sectional study is to determine the effectiveness of osteoporosis screening, diagnosis, treatment, and education in patients who received care at a Midwest level-1 trauma center. Additionally, we aimed to understand the potential barriers that prevent our patients from following through with bone health recommendations.

## Patients and Methods

After institutional review board approval, patients who presented to our orthopedic trauma service were screened prospectively using our electronic medical record system. Patients who sustained fracture(s) regardless of location and mechanism were consecutively enrolled. Patients 55 years of age and older were chosen for their increased risk of fragility fracture. The study was carried out from May 15, 2016 to July 28, 2016; 100 patients participated. The sample size of 100 was chosen to obtain enough observations to be able to reasonably obtain precise estimates regarding the effectiveness of osteoporosis screening and treatment without exceeding the practical resource budget.

After obtaining informed consent, the patients were asked questions from the Fracture Risk Assessment Tool (FRAX; available online at https://www.shef.ac.uk/FRAX/tool.jsp) to determine their FRAX score.^15^ The 12-question osteoporosis screening questionnaire included questions about patient demographics (age, sex, height, and weight) as well as questions regarding personal and family history. The UK version, rather than the US version, of the FRAX assessment tool was used to calculate the 10-year risk for hip and other major osteoporotic fractures because it includes management guidelines (US version does not). The UK version of the tool has been accepted by the International Osteoporosis Foundation for the use in all patient populations due to the treatment recommendations it generates.^16^ The FRAX assessment tool and the National Osteoporosis Guideline Group recommendations^17^ were used to determine the adequacy of osteoporosis management.

**Figure 1.**
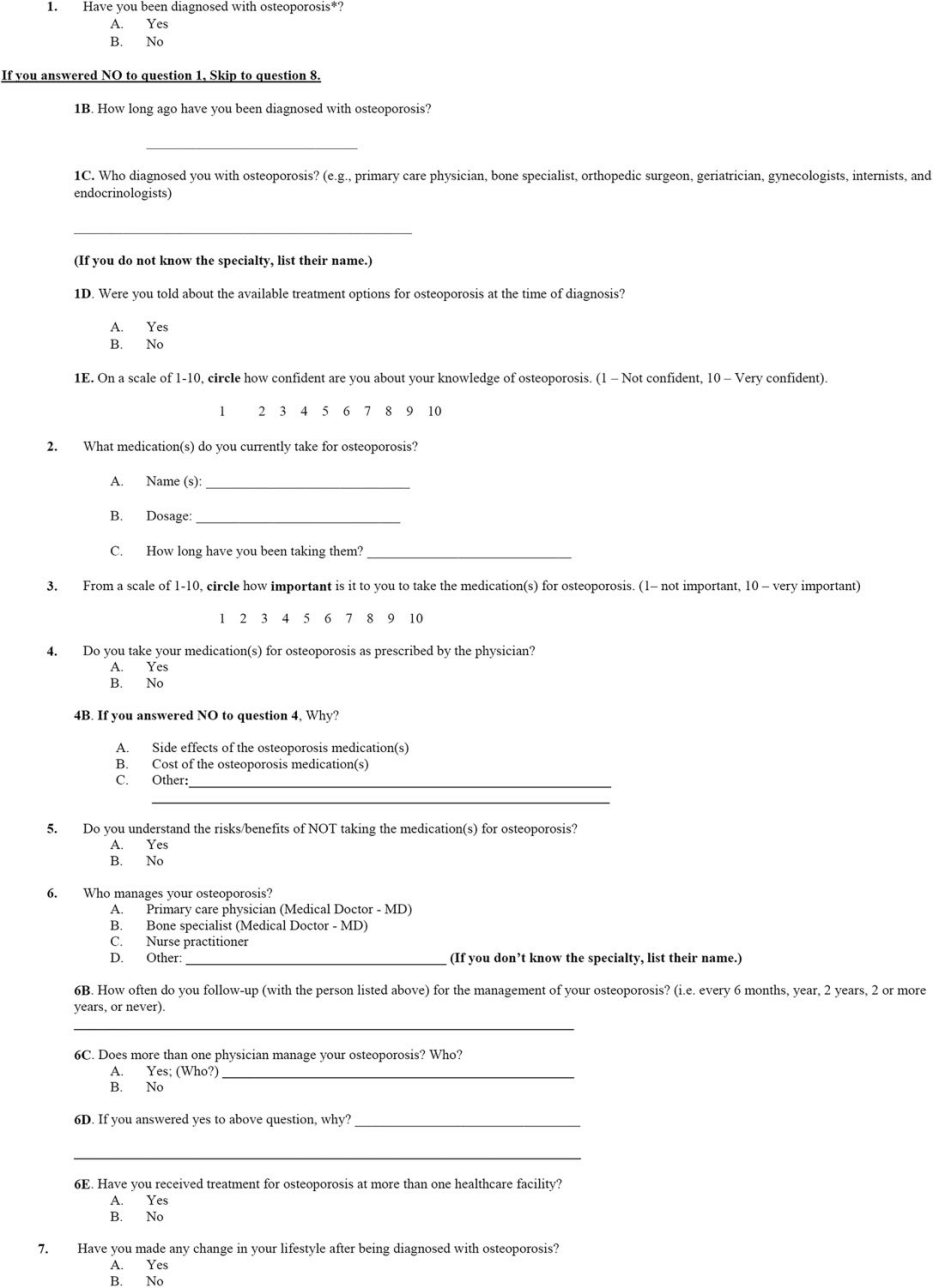

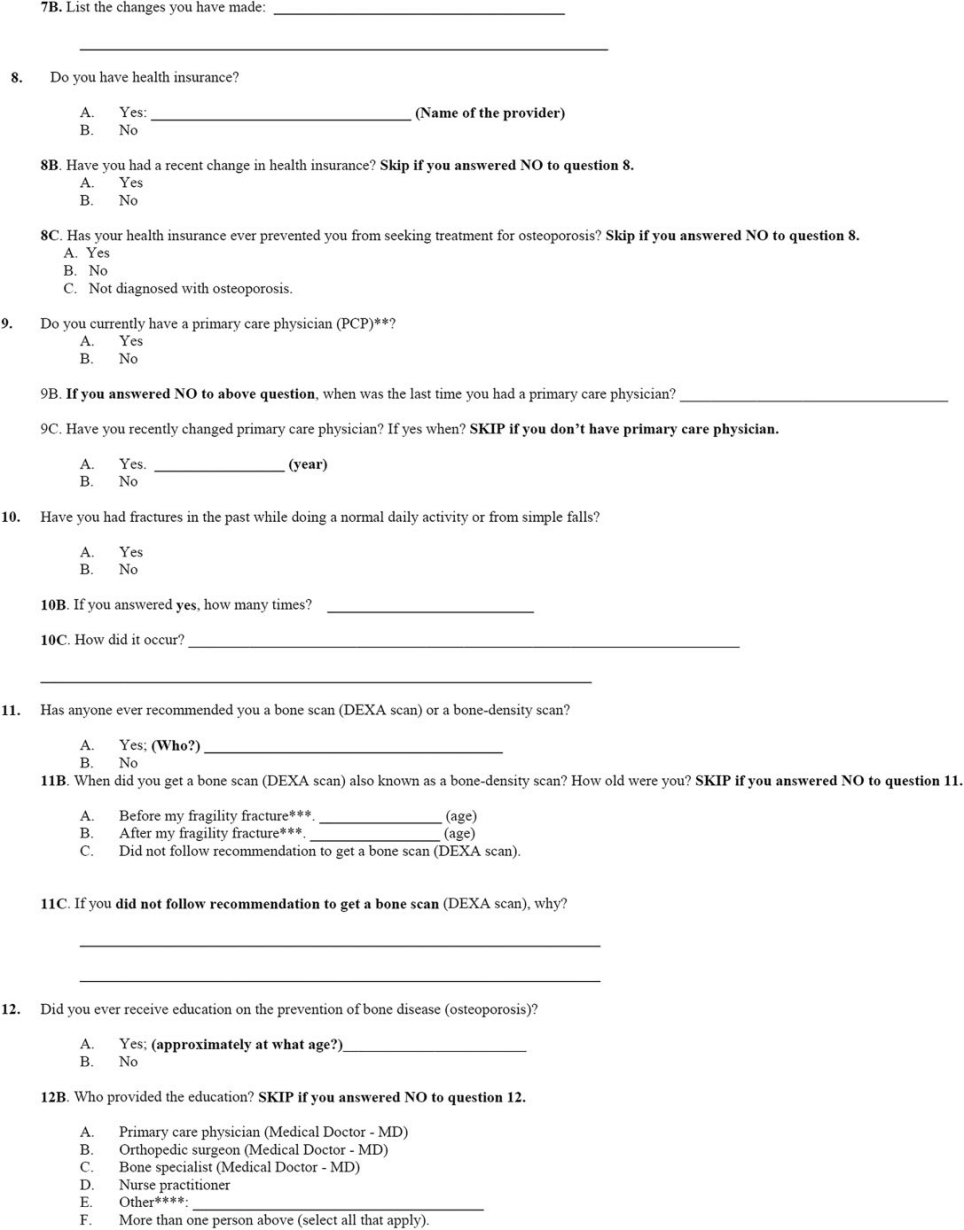
Questionnaire: effectiveness of osteoporosis screening and treatment protocol at the orthopedic trauma center.

Patients were also asked questions about osteoporosis through a study-specific questionnaire that aimed to identify their level of understanding, current and past management, and preventative education. Due to lack of availability of medical records in some patients (ie, new patients initially managed elsewhere), it was decided not to use electronic medical records to determine the history of osteoporosis diagnosis. All patients answered every question of the study-specific questionnaire to the best of their ability. Questions included the status of osteoporosis diagnosis; current treatment, if applicable; the level of understanding about treatment and osteoporosis; preventative education (eg, lifestyle changes); use of DXA screening; and history of fractures with the type of mechanism (Figure 1). Based on their response (yes/no) to the status of osteoporosis diagnosis, patients were separated into 2 groups for evaluation. Group A includes patients who answer “no” to the previous diagnosis of osteoporosis. Group B includes patients who answer “yes” to the previous diagnosis of osteoporosis.

A power analysis was performed using G*Power 3.1.^18^ We determined that a sample size of 100 would be a sufficient number of patients to provide an 80% power with a significance of .05, given an effect size of 0.3. The effect size was calculated to determine a 10% difference in DXA screening recommendation with a standard deviation of 10%. Statistical analyses were performed using SPSS 24 (IBM Corp, Chicago, Illinois). For categorical variables, Pearson χ^2^ and Fisher exact tests were performed. Fisher exact tests were performed on 2 × 2 tables, and Pearson χ^2^ was performed on larger contingency tables. Pearson χ^2^ tests were performed on the following variables: DXA before fragility fracture, DXA screening or diagnosis specialty, and education provider. Fisher exact tests were performed on the following variables: sex, history of fragility fracture, DXA within the past 2 years, DXA previously recommended, taking calcium and vitamin D, and education received. For continuous variables, Levene test for equality and variances was conducted on all continuous variables to determine the equal or unequal variance. Based on the results of Levene test, *t* tests assuming equal or unequal variance were performed. *T* tests were performed on 2 variables: age and number of fragility fractures. A *P* value <.05 was considered statistically significant. Graphs were created with Origin (OriginLab, Northampton, Massachusetts).

## Results

### Demographics

The average age of patients in group A (N = 71), those not diagnosed with osteoporosis, was 66.5 ± 9.2 years (Table 1). The study group consisted of 47 (66.2%) of the 71 females and 24 (33.8%) of the 71 males. The average age of females in the group was 65.9 years, and the average age of males in the group was 67.1 years. The average age of patients in group B (N = 29), those diagnosed with osteoporosis, was 72.7 ± 10.5 years. The group had 25 (86.2%) of the 29 female and 4 (13.8%) of the 29 male distribution. The average age of females in the study group was 72.7 years, and the average age of males in the study group was 72.3 years. Patients who had been diagnosed with osteoporosis were significantly older (72.7 vs 66.5, *P* = .009), and there was a female predominance (86.2% vs 66.2%, *P* = .043).

**Table 1.** Patient Demographics.^a^

	Sex		Age (Mean [SD])
Group	Male	Female	
Group A	24 (33.8%)	47 (66.2%)	66.5 (9.2)
Group B	4 (13.8%)	25 (86.2%)	72.7 (10.5)
*P* Value	.043		.009

Abbreviation: SD, standard deviation.

^a^Group A (N = 71): not diagnosed with osteoporosis; group B (N = 29): diagnosed with osteoporosis.

### Diagnosis

Significantly, fewer patients without the diagnosis of osteoporosis had a history of fragility fracture (56.3%) compared to 92.9% of those diagnosed with osteoporosis (*P* < .001). The majority (72.5%) of patients without a diagnosis of osteoporosis still had a history of fragility fracture, but those patients diagnosed with osteoporosis were more likely to have a history of 2 or more fragility fractures (66.4%). On average, patients without the diagnosis of osteoporosis had 1.3 ± 0.6 fragility fractures, and those with the diagnosis of osteoporosis had 2.0 ± 1.0 fragility fractures on average (*P* = .082; Table 2).

**Table 2.** Patient Screening.^a^

Variable	Category	Group A	Group B	*P* Value
Number of fragility fractures		1.34 ± 0.617	1.96 ± 0.958	.082
History of fragility fracture	Yes	40 (56.3%)	26 (92.9%)	**<.001**
	No	31 (43.7%)	2 (7.1%)	
DXA within past 2 years	Yes	22 (31.0%)	14 (48.3%)	.102
	No	49 (69.0%)	15 (51.7%)	
DXA previously recommended	Yes	36 (50.7%)	25 (89.3%)	**<.001**
	No	35 (49.3%)	3 (10.7%)	
DXA before fragility fracture	Yes	20 (55.6%)	13 (52.0%)	.499
	No	12 (33.3%)	11 (44.0%)	
	Noncompliant	4 (11.1%)	1 (4.0%)	
DXA screening or diagnosis specialty	Orthopedic surgeon	12 (16.9%)	9 (31.0%)	.486
	Primary care	15 (21.1%)	12 (41.3%)	
	Other physicians	4 (5.6%)	6 (20.7%)	
	Nonprofit organization	3 (4.2%)	0 (0%)	
	Unknown	2 (2.8%)	2 (6.9%)	

Abbreviation: DXA, dual-energy X-ray absorptiometry, A P value <.05 was considered statistically significant.

^a^Group A (N = 71): not diagnosed with osteoporosis; group B (N = 29): diagnosed with osteoporosis.

Group A patients were significantly less likely (*P* < .001) to have been previously recommended to undergo a DXA scan (50.7%) when compared to group B (89.3%), regardless of which provider made the recommendation. For those patients who had DXA screening performed, the timing in relation to their fragility fracture was similar (*P* = .499). The noncompliance rate for getting the DXA scan was similar between the groups. However, 62.3% (33 of 53) of patients with 1 or more clinical indications for osteoporosis screening did not receive a DXA scan within the past 2 years, and there was no significant difference between the groups (*P* = .102). For those patients in group B, they were most likely to be diagnosed by their primary care practitioner (PCP; 41.3%).

Based on the survey conducted with this research project, those without the diagnosis of osteoporosis had a FRAX score with an average 10-year major fracture risk of 16.9% ± 10.6% and average 10-year hip fracture risk of 5.6% ± 6.6%. Based on the FRAX score, 28 (42.4%) were recommended to receive DXA scan to measure BMD with lifestyle changes. Another 18 (27.3%) were recommended to have treatment without the need for DXA scan with lifestyle changes, and 20 (30.3%) were recommended to have only lifestyle changes and reassess in less than 5 years based on clinical context. A FRAX score could not be calculated in 5 patients with average body mass index of 54.3 ± 9.6 kg/m^2^.

### Treatment

**Figure 2.**
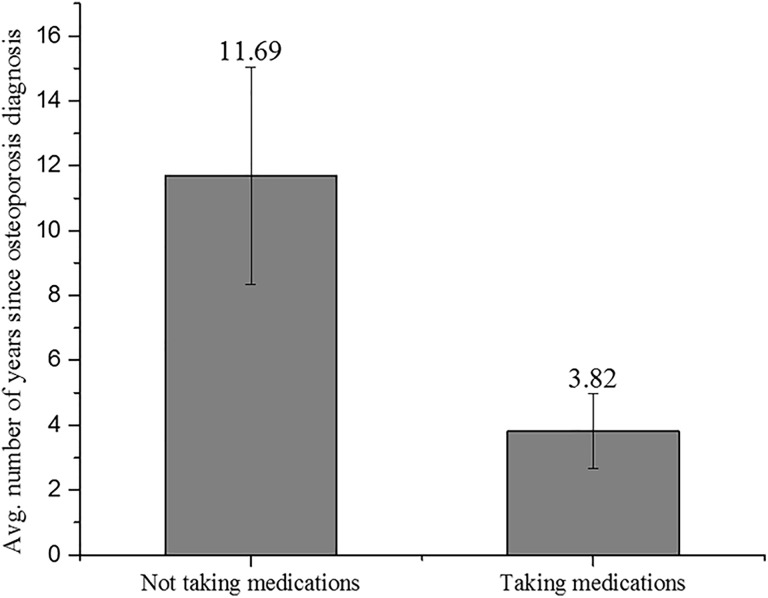
Group B: taking medications versus not taking medications and how long ago they were diagnosed.

Twenty-seven (93.1%) patients diagnosed with osteoporosis were taking calcium and vitamin D supplementation compared to 47 (66.2%) of those without the diagnosis of osteoporosis (*P* = .005; Table 3). Eleven (37.9%) patients in group B, with the diagnosis of osteoporosis, were receiving US Food and Drug Administration–approved medications for the management of their disease. Six (20.7%) reported that they were not prescribed any medications by their healthcare provider(s). Three (10.3%) of the 29 patients were not taking any medication due to a recent diagnosis and reported that they did not have the chance to follow-up with their health-care provider for treatment. Only 1 (3.5%) of 29 claimed that cost was the primary reason for not taking medications, and another 1 of 29 (3.5%) claimed that side effects were the reason for not taking medications. Seven (24.1%) patients had other reasons for not taking the medication or did not answer the question (eg, drug interactions, recent cancer diagnosis, or chose to stop). In retrospect, 6 (20.7%) of 29 patients felt that health insurance was a barrier to receiving appropriate osteoporosis treatment. It was observed that the patients not receiving treatment for osteoporosis were diagnosed on average 11.7 ± 13.8 years ago, while the patients receiving treatment for osteoporosis were diagnosed on average 3.8 ± 4.0 years ago (Figure 2), which was significantly less time since diagnosis (*P* = .038).

**Table 3.** Patient Treatment.^a^

Variable	Category	Group A	Group B	*P* Value
Taking calcium and vitamin D	Yes	47 (66.2%)	27 (93.1%)	**.005**
	No	24 (33.8%)	2 (6.9%)	
Receiving FDA–approved treatment	Yes		11 (37.9%)	
	No		18 (62.1%)	

Abbreviation: FDA, US Food and Drug Administration, A P value <.05 was considered statistically significant.

^a^Group A (N = 71): not diagnosed with osteoporosis; group B (N = 29): diagnosed with osteoporosis.

Nearly half of the patients (35, 49.3%) in group A, those without the diagnosis of osteoporosis, were not receiving treatment and had a FRAX score that indicated a 10-year risk score of ≥3% for hip fractures or ≥20% for major osteoporotic fracture.^19^

### Education

Both groups had a significant percentage of participants claim that they did not receive any preventative education about osteoporosis—group A 83.1% and 72.4% of group B (*P* = .165). Patient education came from similar sources between the 2 groups when it was provided (*P* = .219; Table 4).

**Table 4.** Patient Education.

Variable	Category	Group A	Group B	*P* Value
Understand risks and benefits on noncompliance	Yes	-	19 (65.5%)	-
	No	-	10 (35.5%)	
Received education	Yes	12 (17.9%)	8 (27.6%)	.165
	No	59 (83.1%)	21 (72.4%)	
Education provider	Orthopedic surgeon	0 (0%)	3 (10.3%)	.219
	Primary care	2 (2.8%)	2 (6.9%)	
	Other	6 (8.5%)	3 (10.3%)	
Lifestyle changes after diagnosis	Yes	-	3 (10.3%)	-
	No	-	26 (89.7%)	

^a^Group A (N = 71): not diagnosed with osteoporosis; group B (N = 29): diagnosed with osteoporosis.

In group B, with previous osteoporosis diagnosis, it was observed that 65.5% (19 of 29) of patients understood the risks and benefits of being noncompliant with their treatment plan for osteoporosis. Additionally, only 3 (10.3%) of the 29 patients reported making lifestyle changes—mainly dietary changes—after being diagnosed with osteoporosis.

## Discussion

Our findings suggest that virtually every area of osteoporosis diagnosis, treatment, and education can improve. Both groups had specific deficits and areas for improvement.

### 

#### Group A: Without previous osteoporosis diagnosis

It is evident from our results that the PCP and orthopedic surgeons are the 2 specialties who play a vital role in recommending osteoporosis screening. This is supported by the American Orthopaedic Association’s “Own the Bone” initiative.^20,21^ But many patients in group A did not receive a DXA scan within the past 2 years despite multiple risk factors. This indicates that significant work is required to improve screening protocols. In patients who have not sustained a previous fragility fracture, adding osteoporosis screening questions to a preventative health examination with general physical examination can significantly increase (96%) the rate of osteoporosis screening.^22^

Likewise, patients without a previous DXA scan were less likely to obtain a DXA scan after sustaining a fragility fracture. Although our fracture protocols automatically use daily vitamin D and calcium, and 50 000 IU ergocalciferol weekly for 8 weeks, the lack of DXA screening in this patient group is the major contributing factor that prevented appropriate osteoporosis evaluation from determining whether other medications were required. Antifracture treatments have shown to benefit patients with fragility fractures with or without osteoporosis and decrease their risk of future fractures.^6^ The Clinician’s Guide to Prevention and Treatment of Osteoporosis recommends that patients with 10-year future fracture risk score of ≥3% for hip fracture or ≥20% for major osteoporotic fracture should be screened with a DXA scan to evaluate bone health and subsequently started on treatment to reduce fracture risk.^6^ We found that 49.3% of patients in group A qualified for treatment based on these recommendations but were not receiving treatment. This represents a gap in patient care that must be filled by a multidisciplinary approach to osteoporosis management.

The majority of group A patients (83.1%) claimed to have not received preventative osteoporosis education. The minority of patients who received a preventative education (10.3%) predominantly received it from their PCP. These data suggest that patients are dependent upon their PCP for osteoporosis education before their diagnosis. No group A patients indicated receiving preventative education from an orthopedic surgeon. Among these patients, 15.4% had 2 or more fragility fractures and hence would have been in the care of an orthopedic surgeon for fracture management in the past. Prevention is essential in this population; time must be taken to educate patients about osteoporosis in hopes of reducing patient morbidity and mortality and reduce health-care cost.

#### Group B: Diagnosed with osteoporosis

Patients diagnosed with osteoporosis (group B) were expected to have benefits of treatments that reduce the future risk of fractures compared to patients in group A. However, the data suggest that group B did not have a significant advantage because only 37.9% were being actively treated. The number was lower than expected when compared to previous studies that have shown up to 58% taking appropriate medication.^22^ However, there have been reports of treatment rates of osteoporosis as low as 2% after hip fractures.^23^

The number of patients who were not prescribed an osteoporosis medication (62.1%) was much higher than expected and indicated that the health-care system is ineffective at identifying, screening, and treating patients for osteoporosis. Farmer et al conducted a survey of orthopedic surgeons and showed the majority of them believe that osteoporosis should be treated mainly by PCPs and they are reluctant in prescribing medication for osteoporosis.^13^ This illustrates a potential lack of cooperation and communication between PCP and orthopedic surgeons, as previously reported by the US Surgeon General in 2004.^24^ In a retrospective study, the rate of treatment increased when the patients were recommended treatment postoperatively or if the diagnosis of osteoporosis was noted in the medical files by their orthopedic surgeon. Both circumstances lead to higher treatment odds.^14^ These studies show the importance of effective communication between PCP, orthopedic surgeons, and the patient. Furthermore, fracture liaison services (FLS) have also shown to increase the rate of treatment, evaluation of BMD, treatment initiation, and adherence to treatment.^25–28^ Adherence to medication is closely related to physician follow-up and provider continuity.^29^ This is evident in group B when one considers the time from diagnosis of osteoporosis and the likelihood of taking medication. Patients recently diagnosed (3.81 years) with osteoporosis were more likely to be taking medications than patients diagnosed on average 11.69 years prior.

Our data support that patients significantly lacked education regarding their osteoporosis diagnosis. Although 72.4% claimed that they did not receive education on prevention of osteoporosis, it is plausible that some patients do not recall receiving information about osteoporosis prevention that occurred, and this may have falsely elevated this number. It does show that 1-time education is not enough for a clinically silent diagnosis like osteoporosis. According to Schulman et al, 2 orthopedic office visits can provide a good opportunity to educate patients on osteoporosis that is cost- and time-effective. After education intervention at the initial and 6-month follow-up visit, patients noted improved ability to define osteoporosis, increased understanding of calcium and vitamin D dietary intake, and increased exercise levels.^30^

Limitations of this cross-sectional analysis exist. This study was a single-center cross-sectional analysis, so it is deficient in external validity and may not accurately represent the general population. Also, many of the questions depended on the patients’ ability to recall the dates and times of certain screening procedures. Therefore, some accuracy was lost in the data, and there is a possibility of recall bias. Furthermore, due to lack of availability of medical records in some patients, it was decided not to use previous medical records across both groups to determine the history of osteoporosis diagnosis. The BMD data were not used to determine FRAX 10-year risks scores. However, in most cases, FRAX alone is comparable to FRAX with BMD.^31^ Finally, it was decided to use patients greater than 55 years to evaluate; however, many cohorts in the past have used patients greater than 50 years. Hence, some valuable information could have been lost regarding screening and treatment for osteoporosis in patients between the ages 50 and 54 years.

Continuing to refine clinical protocols with an evidence-based approach is necessary to help patients and alleviate the public and private burden caused by osteoporosis.^1,32,33^ Protocols involving a multidisciplinary approach are useful. Follow-up care and patient education are critical factors in successful osteoporosis management. Since orthopedic surgeons are not likely to continue long-term follow-up of patients sustaining fragility fractures, an FLS has been recommended as an alternative way to ensure long-term follow-up care that avoids placing the sole responsibility on the PCP.^21^

Osteoporosis diagnosis is still an issue, even in patients who have sustained fragility fractures. Osteoporosis screening, management, and education were severely lacking in patients with and without risk factors for osteoporosis. The initial time from diagnosis can play a significant role for patients who continue treatment, and this study emphasizes the need for long-term follow-up for osteoporosis.
